# Long-term clinical and radiological outcomes following non-union surgery: ongoing remodeling and improvement in clinical findings after five years

**DOI:** 10.1186/s13018-026-06991-1

**Published:** 2026-05-30

**Authors:** Jessica C. Böpple, Anas Skeik, Michael Tanner, Thomas Ferbert, Tim Niklas Bewersdorf, Christian Schamberger, Gerhard Schmidmaier, Sebastian Findeisen

**Affiliations:** 1 Clinic for Trauma and Reconstructive Surgery, Center for Orthopedics, Trauma Surgery and Spinal Cord Injury, University Hospital Heidelberg, Schlierbacher Landstraße 200a, 69118 Heidelberg, Germany; 2 Department for Orthopaedics and Traumatology, Theresienkrankenhaus Mannheim, Bassermannstr. 1, 68165 Mannheim, Germany

**Keywords:** Non-union, Long-term outcome, Bone remodelling, Lane-Sandhu-Score, SF-12, Trauma surgery

## Abstract

**Background:**

Non-unions after fractures are known to be a devastating complication in trauma surgery and studies assessing long-term patient-reported, clinical, and radiological outcome measures are scarce. Most evidence-based research on non-unions and their long-term follow-up evaluate the first two post-operative years while excluding subsequent years and their impact on the overall outcome. This study aimed to evaluate the functional, clinical and radiological outcomes five years post-operative.

**Methods:**

In this prospective study, all patients treated operatively between June 2018 and December 2020 for non-unions including every site were systematically reviewed for inclusion and summoned for a follow-up after 5 years. The outcome was evaluated using the modified Lane-Sandhu-Score (LSS), the short-form 12 (SF-12), active range of motion (ROM) and visual analogue scale (VAS). Furthermore, data on complications was collected. For all analyses, a *p*-value < 0.05 was evaluated as significant.

**Results:**

A total of 45 patients were included in the study with a follow-up time of 63 months (IQR: 55; 66). The LSS median after five years was 4 (IQR: 4; 4) and of the VAS 0 (IQR: 0; 4). 95.6% of patients were satisfied with the therapy after five years. Regarding the SF-12, the median score for the mental component was 55.2 (IQR: 48.7; 58.8) and the physical component averaged at 44 ± 10.84. From the second to the fifth post-operative year, patients showed significant improvements in the LSS (Z = − 4.334, *p* < 0.0001). Additionally, improvements in VAS (3 [IQR: 0.5; 4.5] at one year vs. 0 [IQR: 0; 4.5] at five years; *p* = 0.008) as well as quality of life (SF12m median at 1 year 54.05 [IQR: 47.4; 57.05] vs. SF-12 m median at 5 years 56.2 [IQR: 50.7; 57.6, *p* = 0.08]; mean SF-12p at 1 year 42.77 ± 9.15 vs. mean SF-12p at 5 years 45.4 ± 9.91, *p* = 0.0468) were evident. 31% of the patients experienced an improvement in ROM compared to the last follow-up.

**Conclusion:**

This study demonstrates that even beyond the second postoperative year, significant progress in both radiological and clinical findings continues. As a result, patients with non-unions should continue to be examined in special out-patient clinics even after completing the first two years of follow-up.

**Supplementary Information:**

The online version contains supplementary material available at 10.1186/s13018-026-06991-1.

## Background

Due to its biological plasticity, bone is capable of a permanent restructuring and a stimulable repairing process [1]. The dynamic equilibrium between bone formation and bone resorption enables the control over a constantly varying mechanical burden on the tissue [2]. Due to patient-dependent and patient-independent factors, this balance can fluctuate, so that despite thorough research and the optimization of algorithms, non-unions remain a challenging complication after fracture [3]. According to the European Society of Tissue Regeneration in Orthopedics and Traumatology (ESTROT), a non-union is defined as a fracture which cannot heal without further intervention regardless of previous treatment duration [4]. On the one hand, a recent nation-wide study by Mills et. al. [5] was able to revise the incidence of non-unions from a previously wide-held estimate of 5–10% down to 1.9% of all fractures. On the other hand, their treatment coincides with a huge socioeconomic burden, as a review by Kanakaris et. al. [6] revealed costs of £15,566, £17,200 and £16,330 for the respective treatment of humeral, femoral and tibial non-unions during best-case scenarios using a cost-identification query. Revision surgeries require careful planning and a structured post-operative follow-up program to ensure persisting union and manage ongoing symptoms. Even at longer-term follow up, patients report decreased quality of life compared to control populations, especially regarding the physical component [7–9, 18–21]. Additionally, previous trials included the use of joint-specific assessments and were able to prove a remaining functional deficit years after surgery [8, 10].

Standard postoperative follow-ups of approximately 2 years are typically performed to assess clinical and radiological healing. However, clinical observations suggest that improvements—particularly in radiological consolidation and clinical function—may continue beyond this period. An extended observation period of five years therefore allows capturing such delayed effects and may optimize the planning of potential revisions in non-union surgeries [11, 12]. Therefore, we investigated long-term data over 5 years regarding the radiological status to evaluate continuing bone formation or remodeling progresses. Additionally, we assessed the clinical status and quality of life of patients.

## Materials and methods

### Patients

In this prospective follow-up cohort study, we included patients which were treated operatively between June 2018 and December 2020, due to a non-union in our department. Inclusion criteria were a non-union of any bone and a minimum age of 18 at the time of surgery as well as written informed consent.

Patients who received cement spacers as final bone substitution, patients with amputations in the meantime and patients who had to undergo total joint arthroplasty and therefore had the non-union site operatively replaced were excluded from the study.

Regarding the smoking status, in addition to comparing smokers and non-smokers, we included smoking history as a potentially influencing factor. According to Kruk et. al. [13], the optimal cutoff of pack-years of smoking history for increased risk of non-union is set at 6.1 pack-years, according to which we additionally separated the patient collective.

The study was approved by the ethical board of the university and the study was conducted in accordance with the Declaration of Helsinki. All patients gave their written informed consent before inclusion.

### Surgical technique

Surgical procedures were performed as one- or two-step procedures, depending on the type of non-union, and strictly following the principles of the diamond concept for successful bone healing. The one-step technique was employed in cases where the defect was minor or in cases where an infection was unlikely. The two-step procedure was utilized when infection was present, when the defect was avascular or large in size in order to improve vascularisation. During one-step procedures, a debridement of the non-union and its surrounding avital tissue was done, subsequently a de-novo osteosynthesis using a suitable and biomechanically stable implant and finally the augmentation of the defect using an autologous bone and/or a bone substitute. In the two-step procedure patients had undergone a total implant removal and debridement of the non-union site with subsequent filling of the bone defect with a polymethyl-methacrylat (PMMA) spacer impregnated with gentamycin/vancomycin in the first step as well as a reosteoynthesis. After six to eight weeks during the second step the spacer was removed and replaced with autologous bone graft and/or a bone substitute. In case of a proofed infection, revisions of the first step until reaching total sterility were performed.

Due to the long inclusion time, a variety of bone substitute materials, partly in addition to an autologous bone-graft, came to use. They consisted of Vitoss, Vitoss BA(2X), tricalcium phosphate (TCP), bioactive glass and cerament genta.

### Post-operative assessment and radiographic evaluation

Clinical assessment consisted of a clinical examination including the neighbouring joints range of motion (ROM). Furthermore, the short form-12-questionnaires (SF-12) [14] and visual analogue scale (VAS) [15] were used to assess the current quality of life as well as pain. Detailed surgical, as well as general patient history was individually reconstructed by consultation during follow-up, from digital records and by using questionnaires from previously carried out studies.

**Fig. 1:**
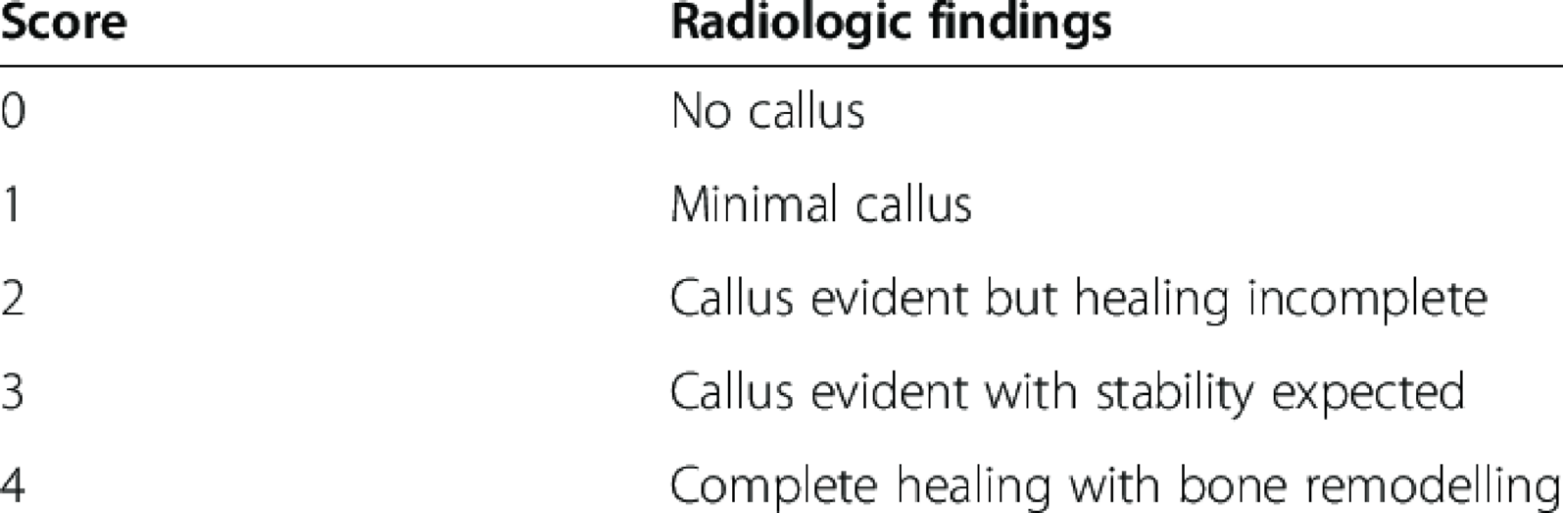
Modified Lane- Sandhu- Score [17]

Radiological assessment was performed with standard radiographs with anterior- posterior and lateral views of the respective body part and utilizing the modified Lane-Sandhu-Score (LSS, Fig. 1) to describe bone healing, bone cortex and bone remodelling. A bone was considered adequately stabilized when reaching a LSS of 3 and fully consolidated when reaching a LSS of 4 [16]. The LSS was determined by an experienced orthopaedic surgeon. Additionally, X-rays and, if available, CT-scans from previous follow-ups were assessed.

### Statistical analysis

Analysis was done using the Statistical Program for Social Science (SPSS) Version 29.0 (IBM Corp., Armonk, NY, USA) and Stata statistical software (version 18.0, StataCorp, United States). Data were described using means and standard deviations or medians and interquartile ranges (IQR), as appropriate. Absolute numbers and percentages were used to illustrate distributions. The Shapiro–Wilk-Test was used to identify normally distributed data. Paired t-tests were used to compare normally distributed data at one- and two-year follow-ups with five-year follow-ups. Data without normal distribution was compared using the Wilcoxon Signed-Rank Test. The influencing factors were analysed using unpaired t-tests against a set of variables collected at five-year follow-up. Furthermore, the Mann–Whitney-U-Test was performed to analyse differences between groups where appropriate and the Wilcoxon-Sign-Rank-Test to analyse the improvement of the LSS over time. For all analyses, significance was set at *p *< 0.05.

## Results

### Study population

**Fig. 2:**
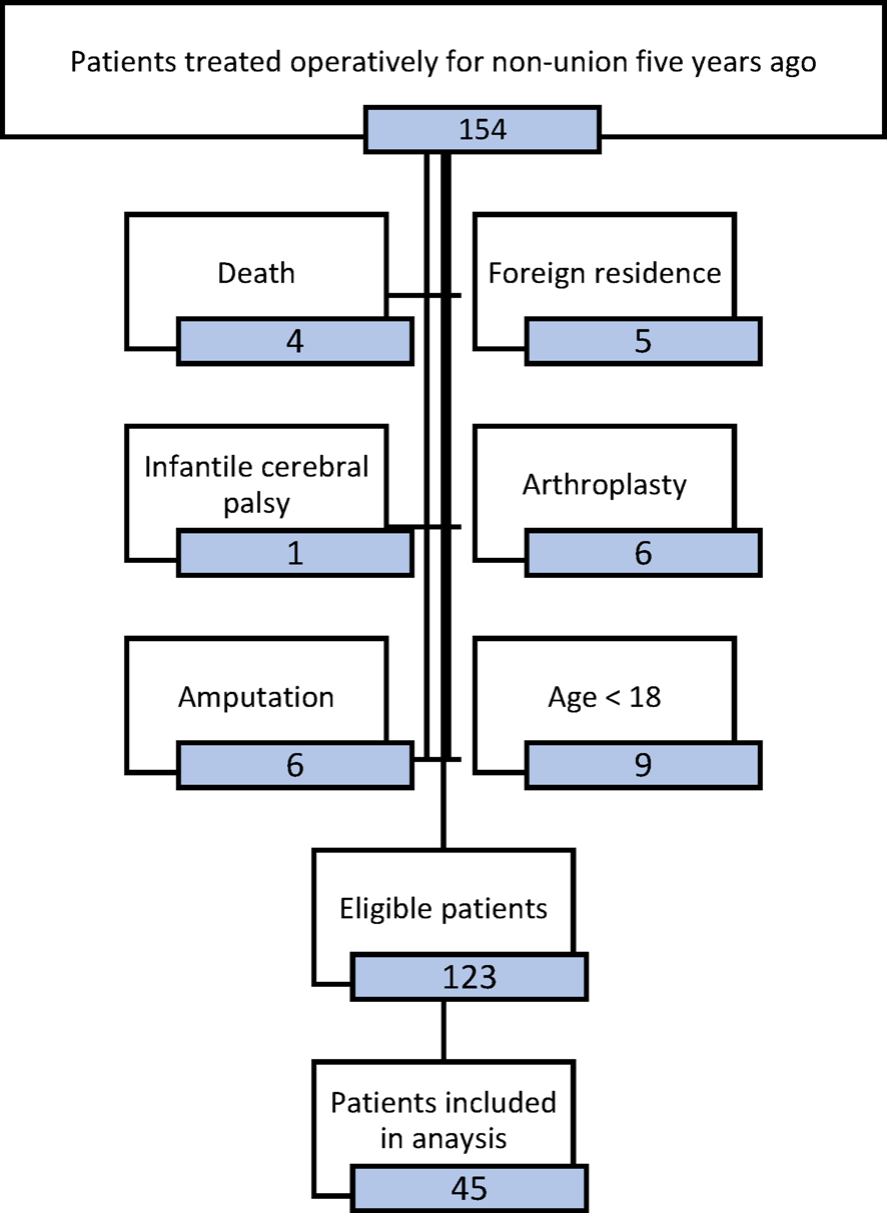
Screening process

Of the 154 patients who underwent surgery in our department between June 2018 and December 2020, thirty-one were excluded from the analysis due to the following reasons: Five of these patients could not be contacted due to foreign residence. Further reasons included amputation, death, arthroplasty, infantile cerebral palsy and an age of less than 18 at the time of surgery. A significant number of patients underwent treatment with long distances from their residence, both nationally and internationally. Few of them were willing to show up for a single long-term follow-up, especially if they were symptom-free. Due to the long-term design of the study, several patients had changed their contact details which sometimes could not be accessed. Out of the remaining 123 patients, 45 patients were recruited and prospectively examined for follow-up at our out-patient clinic between June and December 2024. The screening process is illustrated in Fig. 2. The median follow-up time was 63 months (IQR: 55; 66).

**Fig. 3:**
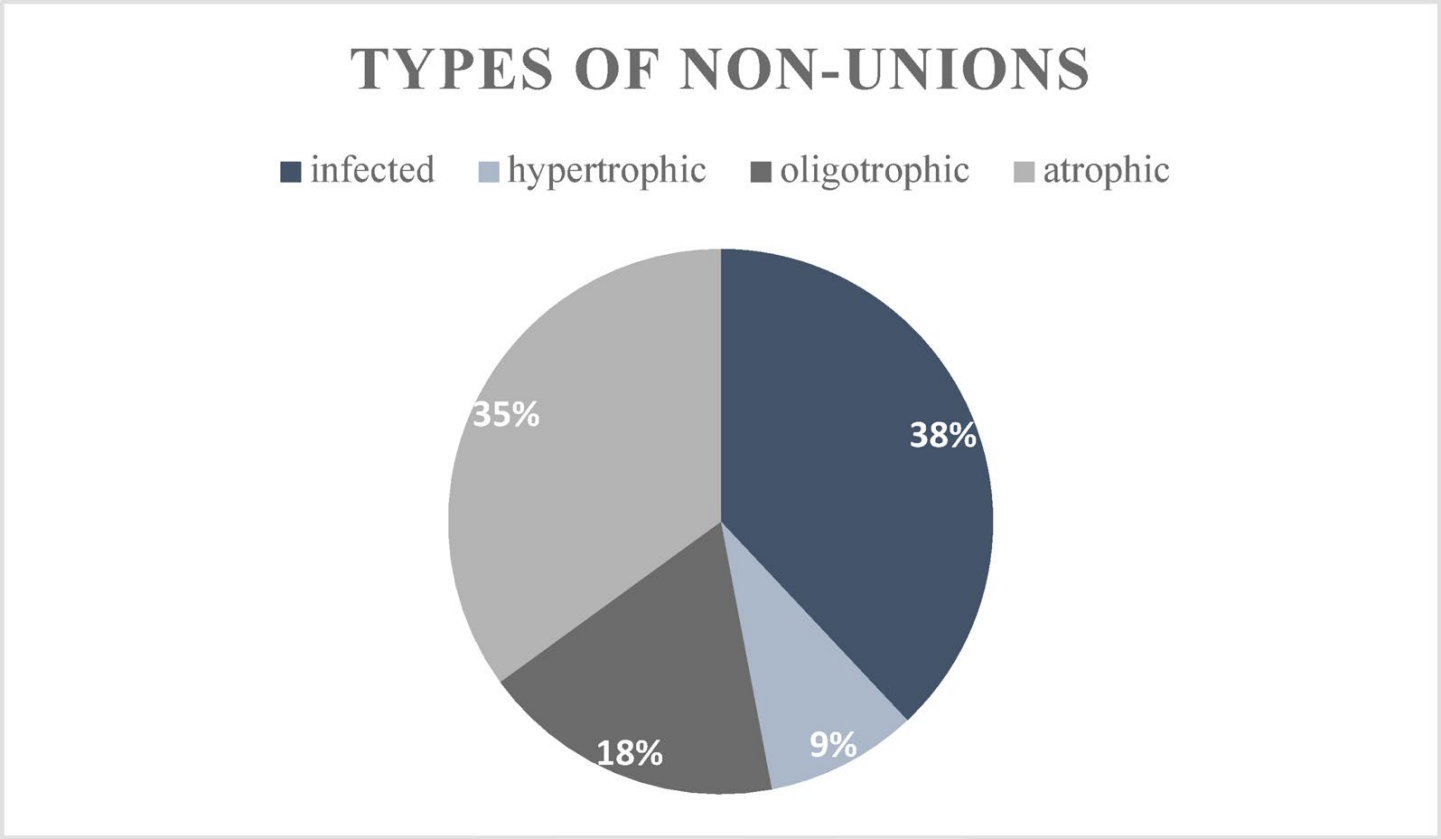
Representation of the distribution of non-union types using a pie chart

A total of 45 patients, with a median age of 55 years (IQR: 46; 65), were included in the study; 27 were male and 18 were female. Twenty-four patients had non-unions of the lower extremity long bones, 11 non-unions were of the upper extremity long bones and ten patients had a non-union of the foot bones. 12 patients had a smoking history of over 6.1 pack-years. Total or partial implant removal was done in six cases. Time between non-union surgery and implant removal averaged at 29 months (range 5–48 months). Table 1 provides a comprehensive overview of the patient characteristics of the entire collective examined. A more comprehensive list of non-unions can be found in Supplementary Table 1. Furthermore, Fig. 3 provides additional information about the type of non-union.

**Table 1:** Patient characteristics (IQR = Interquartile Range)

Factor	Value
Age, median (IQR) [years]	55 (46; 65)
Gender	
female	18 (40%)
male	27 (60%)
Infectious non-union	
yes	17 (38%)
no	28 (62%)
Cause of fracture	
Open	8 (18%)
Closed	26 (58%)
Iatrogenic	
Type of surgery	
One-step	15 (33%)
Two-step	30 (67%)
Smoker	
Yes (active)	8 (18%)
No	
Former	11 (24%)
Never	26 (58%)
Smoker with	
> 6.1 pack-years	12 (27%)
never or < 6.1 pack-years	33 (73%)
Time to surgery in months, median (IQR)	19 (13.5; 31.5)
Follow-up in months, median (IQR)	63 (55; 66)
Additional surgeries, median (IQR)	3 (1.5; 4.5)
Autologous bone-graft	
Yes	32 (71%)
No	13 (29%)
Vitoss	
Yes	14 (31%)
No	31 (69%)
Bioglass	
Yes	23 (51%)
No	22 (49%)
Diabetes	
Yes	5 (11%)
No	40 (89%)
Localisation	
Humerus	5 (11%)
Clavicle	1 (2%)
Forearm	5 (11 %)
Femur	6 (13%)
Tibia	17 (38 %)
Ankle joint	4 (9 %)
Hindfoot	4 (9 %)
Midfoot	1 (2 %)
Forefoot	2 (5 %)

With regard to the long tubular bones, the distribution of infected non-unions was as follows: 1 × in the humerus, 2 × in the radius, 2 × in the femur and 11 × in the tibia. Regarding the non-infected non-unions of the long bones, the humeral non-unions exhibited a marked atrophic tendency according to the classification proposed by Weber/Cech. The radial non-unions were both atrophic and oligotrophic. Five out of six non-unions in the femur were atrophic. The majority of non-unions in the tibia (7 out of 18) were also atrophic. The precise distribution is illustrated in the Supplementary Table 2.

### Radiological Outcomes

In the present study, three of the 45 patients (6.7%) were excluded from the evaluation of radiological results. This was due to the necessity of revising their cases prior to the two-year follow-up, owing to treatment failure.

After five years, the median LSS for the remaining 42 patients without further interventions was 4 (IQR:4—4). 36 out of 42 patients (85.7%) in total achieved an LSS of 4 and two patients (4.8%) an LSS of 3. This transcends to an overall union-rate of 90.5% and a total of four (9.5%) persisting non-unions for the included 42 patients.

Thirty-six patients (85.7%) out of the 42 patients included for the radiological analysis had x-rays done in their second postoperative year. Detailed distribution of the LSS can be found in Table 2.

**Table 2:** modified LSS distribution after 2 years (n = 36) and 5 years (n = 42) for patients without further interventions (LSS = Lane-Sandhu-Score)

LSS	2 years, N (%)	5 years, N (%)
0	1 (2.8)	0 (0)
1	2 (5.6)	1 (2.4)
2	4 (11.1)	3 (7.1)
3	14 (38.9)	2 (4.8)
4	15 (41.7)	36 (85.7)

The two-year follow up Lane-Sandhu-Scores were analysed against those five years post-operative using a Wilcoxon signed-rank test resulting in a significant difference between the two measurements (Z = − 4.334, *p* < 0.0001).

**Fig. 4:**
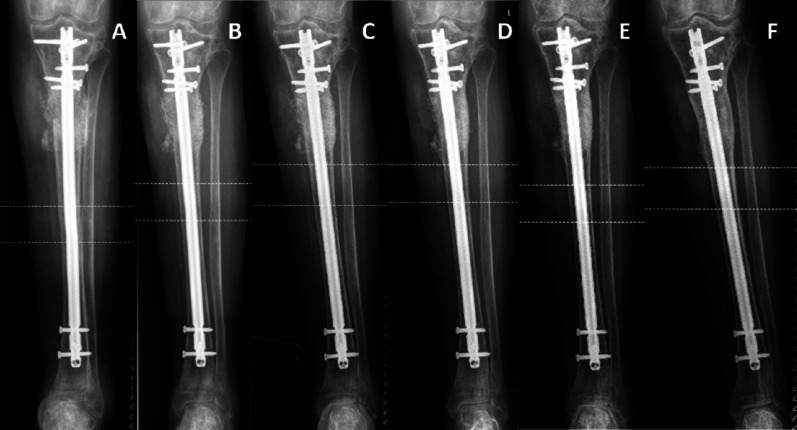
Example of a non-union during a 5-year follow-up; Post-operative X-rays after **A** six weeks **B** twelve weeks **C** six months **D** one year **E** two years (F) five years

An example of a complete radiological follow-up of a patient with a non-union of the proximal tibia is displayed in Fig. 4.

Patients who did not achieve an LSS of 3 or greater after a 5-year period were found to have non-unions in various locations. These include humerus, forearm, tibia as well as the foot.

The median LSS for the various non-union localications are displayed in Table 3, alongside the union rate.

**Table 3:** Median of the modified Lane-Sandhu-Score as well as union rates shown for the various locations

Localisation	Clavicle N = 1	Upper arm N = 4	Lower arm N = 5	Upper leg N = 6	Lower leg N = 16	Foot N = 10
LSS (Median; IQR)	4 (4; 4)	4 (1,75; 4)	4 (3; 4)	4 (3,75; 4)	4 (4; 4)	4 (4; 4)
Union rate (%)	100	75	80	100	93,75	90

Using the Kruskal–Wallis test no significant differences between the different non-union locations regarding the time to consolidation could be found with *p* = 0.5905. The exact times of consolidation can be found in Table 4.

**Table 4:** Time of consolidation in months for the non-unions who reached osseous consolidation with a lane-Sandhu-score (LSS) ≥ 3 (IQR = interquartile range)

	Clavicle N = 1	Upper arm N = 3	Lower arm N = 4	Upper leg N = 6	Lower leg N = 15	Foot N = 9
Time to union [months], *median (IQR)*	12 (12; 12)	6 (6; 60)	6 (6; 15)	12 (12; 24)	12 (12; 24)	12 (6; 12)

An overview of all patients with an LSS below 4 at the 5-year follow-up and potential risk factors is provided in Supplementary Table 3.

### Clinical outcomes

In total, 43 patients (95.6%) stated they were satisfied with the overall treatment at the five-year follow-up. The median visual analogue scale score at five-year follow-up was 0 (IQR: 0; 4) points for the entire cohort with 23 patients (54.8%) reaching a score of 0. Twenty-one patients (45.7%) had a VAS stated at one-year follow-up. This group showed a median VAS of 3 (IQR: 0.5; 4.5) points at one year. After five years, Wilcoxon Sign-Rank Test showed a significant improvement as VAS decreased to a median of 0 (IQR: 0; 4.5) points for this group, indicating an improvement in pain levels over the additional follow-up period (*p* = 0.008).

Regarding the quality of life, SF-12 scores for the entire cohort had a median SF-12 m of 55.2 (IQR: 48.7; 58.8) points and an average SF-12p of 44 ± 10.84 points. Twenty-two patients had available data for quality of life at one-year follow-up. Their median mental component score was 54.05 (IQR: 47.4; 57.05) and their mean physical component score was 42.77 ± 9.15. Regarding the mental component score, the Wilcoxon Signed-Rank Test showed an improvement by the five-year follow-up without statistical significance, as scores for this group reached 56.2 (IQR: 50.7; 57.6, *p* = 0.08). Paired sample t-tests revealed a significant improvement of the physical component score by the five-year follow-up, as scores for this group reached an average of 45.4 ± 9.91 (*p* = 0.0468).

Thirteen patients (31%) showed an improvement in ROM of the neighbouring joints at five-year follow-up in comparison to the most recent examination.

### Influence of common risk factors

No significant difference between smokers and non-smokers regarding the LSS was found using the Mann–Whitney-U-Test (Z = 0.418, *p* = 0.6761) with a median of 4 (IQR: 3,25; 4) and median of 4 (IQR: 4; 4) respectively. Non-smokers had insignificantly higher median scores in both physical (45.5 (IQR: 35.5; 52.95) vs. 45.05 (IQR: 29.15; 53.8), *p* = 0.814) and mental (55.2 (IQR: 50.4; 58.8) vs. 55 (IQR: 35.3; 59), *p* = 0.694) quality of life as well as a lower median VAS (0 (IQR: 0; 3) vs. 2 (IQR: 0; 6.75), *p* = 0.403) at five-year follow up than smokers.

No significant difference between patients with a pack-year number of 6.1 or higher regarding the LSS was found using the Mann–Whitney-U-Test (Z = -0.298, *p* = 0.766) with a median of 4 (IQR: 4; 4) and median of 4 (IQR: 4; 4) respectively. When examined solely for the presence of consolidation and pack-year status with < or > 6.1 years, no substantial disparities were detected in the chi-square test with *p* = 0.868. Patients with a pack-year number of 6.1 or higher have exhibited a lower median physical quality of life (44.5 (IQR: 29.15; 46.05) vs. 48.85 (IQR: 36.63; 55.45); *p* = 0.11) and a higher VAS (3.5 (IQR: 0; 6.75) vs. 0 (IQR: 0; 3); *p* = 0.098), latter being marginally significant. The scores for mental quality of life were similar for patients with a pack-year number above or below 6.1 (55.7 (IQR: 37.55; 59) vs. 55.2 (IQR: 48.73; 58.8); *p* = 0.837).

In regards to infections, no difference was found regarding the mental component of the SF-12, as scores reached a median of 55.3 (IQR: 49.2; 57.6) for the patients with infectious non-unions and 55.1 (IQR: 47.3; 58.8) for the patients with non-infectious non-unions (*p* = 0.927). Patients without infectious non-unions had higher median scores for the physical component of the SF-12 (SF-12p = 46.3 (IQR: 33.7; 55.9) vs. 44.6 (IQR: 36.1; 50.3), *p* = 0.294) and lower median VAS scores (0 (IQR: 0; 3) vs 3 (IQR: 0; 4); *p* = 0.198) than patients with infectious non-unions. Infectious non-unions have exhibited a higher Lane-Sandhu-Score than non-infectious non-unions without statistical significance (Z = − 1.122, *p* = 0.2618).

### Post-operative complications

Six patients (13%) required reinterventions: three patients for persisting non-union, two for persisting infection (12% of initially infectious non-unions) and one due to the remains of cement spacer previously implanted causing pain. The three revisions due to non-union were performed using bioactive glass in all cases and additional autologous bone-graft in one case. The mean time between non-union surgery and revision was 8 ± 3.5 months.

In both cases with persisting infection, a simple wound debridement and oral antibiotic therapy was sufficient. In the case with the cement spacer remains, a removal of this residual could be performed successfully.

In two different patients, intra-operative samples were positive for staph. Epidermidis. Due to high suspicion of contamination, they were not counted as post-operative complications. In both cases oral antibiotic therapy was sufficient.

## Discussion

The aim of this first-of-its-kind study was to evaluate the status of our patient collective five years after non-union surgery and highlight the progress observed between the second and fifth post-operative year.

The union-rate reached 90.5% and significant improvements were noted from the one- and two-year follow-ups to the five-year follow-up in terms of SF-12p, VAS and LSS. These results suggest that, even after two years post-operatively, significant continued progress in these variables can be expected. However, the precise timing and nature of the osteogenesis and remodeling’s most significant changes remain uncertain, particularly whether these changes will occur within the period between year two and five. Regarding influencing factors, none were found to have a significant influence on patient’s outcomes, although a smoking history of more than 6.1 pack-years emerged as the factor with the highest impact, with a marginally significant negative effect on the VAS scores.

While there is little research surrounding the long-term success of non-union surgery in general, none of these none of these studies have addressed the exact 5-year outcomes following non-unions surgeries including all non-union sites to best of our knowledge. However, several studies have analyzed the long-term outcomes of lower limb non-union surgery although these were not confined to a specific point in time. For example, Poutoglidou et. al. [18] investigated 77 patients with lower limb non-unions with a mean follow-up of 41.41 months. It differentiates from our study through the inclusion of conservatively treated patients and the focus on lower limbs. Despite this, a union-rate of 91% could be observed, which is in line with our findings. Similarly, Wagner et. al. [8] analyzed the long-term outcome of 124 patients following lower limb non-union surgery with an average follow-up of 8.6 years, reporting a union rate of 75%. The inclusion of patients operated on from 2001 until 2021 could attribute to the lower union-rate, as treatment algorithms, post-operative care and the optimization of comorbidities have evolved since then.

Looking at the pain assessment, Wang et. al. [19] analyzed the VAS in 20 patients who underwent non-union surgery after intramedullary nailing and bone grafting of lower limb fractures 36.3 months after the final surgery, resulting in a mean VAS of 0.75 (± 0.96). This is consistent with our findings, which showed a median VAS score of 0 after five years. One possible explanation for a possibly overall lower VAS, given their standard deviation and our IQR, is that some of our patients might have experienced more pain at 5 years-follow-up. This could be due to the difference in the number of surgeries performed, as patients in their study underwent one initial fixation surgery and one additional non-union surgery, whereas in our study, the median number of surgeries was 5 (IQR: 3; 6.5). This higher total number of surgeries could be due to the greater variety and complexity of cases in our study, which may also contribute to a higher VAS in some patients. In comparison to that, Fisher et. al. [20] evaluated the long-term outcomes of patients after non-union surgery at an average follow-up of 24 months and found an average VAS-score of 2.77. One can assume that this is in line with our results, as the shorter follow-up period is a possible explanation for the higher VAS-score. Other studies have used different scores to measure pain. In a long-term study about aseptic long bone non-unions, Walter et. al. [7] divided an instrument of quality-of-life measurement, the EQ-5D, into its subdimensions and found that after a mean of 4.7 years after surgery, 80.3% of patients still dealt with pain or discomfort. This high percentage could be due to the EQ-5D being a more general instrument, as it includes discomfort and can include pain which is not correlated to the surgery.

As for quality of life, several studies have discovered an improving, however still impaired quality of life 12 months after surgery [21–24], with little data existing beyond the one-year follow-up. Zeckey et. al. [25] compared patients with femoral and tibial non-unions to patients with uneventful fracture healing of the respective bone regarding their quality of life using the SF-12. Patients operated on between 2000 and 2008 were included in this study from 2011, however an average follow-up time was not clarified. The result was a significantly lower physical score for both locations and a significantly lower mental score for the femoral non-unions only. Furthermore, Wagner et. al. [8] assessed patients’ long-term quality of life using the SF-12, resulting in a median SF-12p score of 42.8 (IQR: 32.7; 52) and a median SF-12 m score of 50.0 (IQR: 41.3; 55.4) after a follow-up of 8.6 years, which are in concordance with our findings. In addition to that, studies assessing patients’ quality of life using the more extended SF-36 have found equivalent results. [7, 26–28]

Similar to our study, Boadi et. al. [29] compared patient reported outcomes at one and eight years on average including all locations and assessed the progress in ROM. An improvement of 56% of patients’ ROM could be detected. However, the statistical power of this figure is limited, as only 16 (26%) of their 62 patients in total could be evaluated regarding ROM. Because most other studies focus on specific locations, the use of joint-specific instruments to measure patients’ mobility becomes preferable, hence making a comparison of ROM assessment across multiple studies challenging.

In terms of smoking, medium-term follow-ups until one year post-operative have shown a significant impact of active smoking on healing, quality of life, and pain scores [19, 30, 31]. At long term however, studies have revealed a lower difference between smokers and non-smokers regarding union rate [32], and a persisting difference regarding pain scores. For example, Fisher et. al. [20] discovered a significantly higher VAS-score of patients who were either active smokers or had smoking history, regardless of the pack-year number. However, patients with smoking history can vary heavily regarding their pack-year number and therefore probable inflicted tissue damage. An introduction of a pack-year cut-off is therefore a reasonable approach, which Kruk et. al. [13] followed in a study identifying ACDF fusion risk factors and found the ideal cut-off to be at 6.1 pack-years. Patients above this cut-off have shown significantly higher risks of non-fusion. Similarly, patients in our study with a pack-year number above this cut-off point were found to have higher VAS scores approaching significance. One potential explanation for the absence of significance could be that the patients with > 6.1 pack-years in our study comprised only 12 patients, which precluded the assumption of a normal distribution. In a larger group with a normal distribution, a significant difference is a conceivable outcome. Despite this, discussing this cut-off when analyzing smoking history as an influencing factor after non-union surgery becomes plausible.

Regarding reinfection rate, Giovanoulis et. al. [33] evaluated 19 patients with infectious non-unions after induced-membrane technique surgery. A similar study with 23 patients was conducted by Liu et. al. [34]. Maini et. al. [35] pursued the same goal in 30 patients with infectious non-unions treated with the Ilizarov method. The rate of recurring infections in all studies after the final surgery ranged from 8.7% to 10.5%, which falls in line with our results.

The higher LSS of patients with infectious non-unions could be attributed to their distinct treatment protocols, as they typically undergo multi-step procedures to ensure asepsis and to facilitate the development of the masquelet membrane as an additional stimulator for perfusion of the site and as an anti-infectious factor.

In summary, although a significant improvement in the LSS was observed between postoperative years two and five, revision surgery should be considered in patients who demonstrate persistent non-union two years after surgery and who either experience pain during full weight-bearing or have not achieved full weight-bearing although the existing implant might be radiologically intact. Patients should generally remain under clinical and radiographic follow-up until radiological consolidation and an acceptable clinical outcome are achieved. Importantly, this does not necessarily imply complete absence of pain or full restoration of function. In our cohort, symptoms, particularly in the lower extremities, improved significantly but did not fully resolve. Notably, functional improvement was not limited to the first two postoperative years but continued in the subsequent years.

Our study has several limitations. For one, the use of x-rays as an imaging tool might be insufficient to fully evaluate the integrity and remodeling processes of the bone in some cases. The use of a CT-scan might be preferable. This issue coincides with the limitations of the LSS, which can describe the presence of bone remodeling but not its extent. Using a more detailed score to better describe the bone remodeling process may address this issue. Moreover, a significant number of patients from our nationwide and international patient pool were unable to attend due to long travel distances. Due to the inclusion of non-unions across all bones and hence the lack of clinical comparability between the patients, no joint-specific assessment test during the follow-up could be performed. Furthermore, only about half of the patient collective had available data for one- or two-year follow-ups, which led to a less compelling comparison between the two time points.

## Conclusion

In conclusion, in this unique follow-up study improvements in both radiological and clinical outcomes can be observed at the five-year follow-up. Based on the results of this study we recommend a follow-up plan that extends after the first two post-operative years, as significant progress continues beyond this period. We believe that our study provides important and innovative discoveries regarding this progress and functions as a foundation for future research to further evaluate the long-term outcome of non-union surgery.

## Supplementary Information

Below is the link to the electronic supplementary material.

Supplementary Material 1

## Data Availability

The datasets used and/or analysed during the current study are available from the corresponding author on reasonable request.

## Abbreviations

ACDF: Anterior cervical discectomy and fusion
ESTROT: European society of tissue regeneration in orthopedics and traumatology
IQR: Interquartile range
LSS: Lane-Sandhu-Score
ROM: Range of motion
SF-12: Short-form 12 questionaire
SPSS: Statistical Program for Social Science
TCP: Tricalcium phosphate
VAS: Visual analogue scale
