# Supplementary material for: Long-term clinical and radiological outcomes following non-union surgery: ongoing remodeling and improvement in clinical findings after five years
Source: J Orthop Surg Res. 2026 May 30;21:317. doi: 10.1186/s13018-026-06991-1 (PMC13224682; doi:10.1186/s13018-026-06991-1)
Supplement: Supplementary file 1 — Supplementary Material 1 [file 13018_2026_6991_MOESM1_ESM.docx]

**Supplements**

| Localisation | Value | Localisation | Value |
| --- | --- | --- | --- |
| **Upper Extremity**  **Clavicle**  Medial  Shaft  Lateral  **Humerus**  Proximal  Shaft  Distal  **Ulna**  Proximal  Shaft  Distal  **Radius**  Proximal  Shaft  Distal | **11**  **1**  0  1  0  **5**  1  3  1  **1**  0  1  0  **4**  1  0  3 | **Lower Extremity**  **Femur**  Proximal  Shaft  Distal  **Tibia**  Proximal  Shaft  Distal  **Fibula**  Proximal  Shaft  Distal  **Foot Bones**  A. talocruralis  Proc. anterior calcanei  A. talonaviculare  A. Tarsometatarsale 1  A. metatarsocuneiforme 1  Os Metatarsale 1  A. Metatarsophalangeale 1 | **24**  **6**  1  2  3  **18**  6  4  8  **0**  0  0  0  **10**  3  1  2  1  1  1  1 |

Supplementary table 1: Table providing a detailed description of all non-union locations

| Localisation | Value | Infectious Non-Unions |
| --- | --- | --- |
| **Humerus**  Atrophic  Oligotrophic  Hypertrophic  **Radius**  Atrophic  Oligotrophic  Hypertrophic  **Femur**  Atrophic  Oligotrophic  Hypertrophic  **Tibia**  Atrophic  Oligotrophic  Hypertrophic | **5**  3  1  1  **4**  2  2  0  **6**  5  0  1  **18**  7  6  5 | **1**  0  0  1  **2**  1  1  0  **2**  2  0  0  **11**  2  5  4 |

Supplementary table 2: Accurate representation of infected vs. non-infected long bone non-unions classified according to Weber-Cech-classification

| Patients number | Age  [years] | Smoker status | diabetes | Open fracture | Infected non-union |
| --- | --- | --- | --- | --- | --- |
| 1 | 71 | Active, 100 py | Yes | Open | Yes |
| 2 | 76 | Active, 70 py | No | Closed | No |
| 3 | 36 | Former, 3.5 py | No | Closed | No |
| 4 | 51 | Never | No | Closed | No |
| 5 | 26 | Never | No | Closed | No |
| 6 | 62 | Never | No | Iatrogen | No |
| 7 | 58 | Never | No | Open | No |

Supplementary table 3: Presentation of all patients with an LSS below 4 at the 5-year follow-up and potential risk factors (LSS= Lane-Sandhu-Score)
